# Ultimate Grounding of Abstract Concepts: A Graded Account

**DOI:** 10.5334/joc.214

**Published:** 2022-03-11

**Authors:** Tim Reinboth, Igor Farkaš

**Affiliations:** 1 Faculty of Philosophy and Education, University of Vienna, Wien, AT; 2 Faculty of Mathematics Physics and Informatics, Comenius University in Bratislava, Bratislava, SK

**Keywords:** embodiment, grounded cognition, graded abstractness, language, conceptual metaphor

## Abstract

Abstraction, one of the hallmarks of human cognition, continues to be the topic of a strong debate. The primary disagreement concerns whether or not abstract concepts can be accounted for within the scope of embodied cognition. In this paper, we introduce the embodied approach to conceptual knowledge and distinguish between embodiment and grounding, where grounding is the general term for how concepts initially acquire their meaning. Referring to numerous pieces of empirical evidence, we emphasise that, ultimately, all concepts are acquired via interaction with the world via two main pathways: embodiment and social interaction. The first pathway is direct and primarily involves action/perception, interoception and emotions. The second pathway is indirect, being mediated by language in particular. Evidence from neuroscience, psychology and cognitive linguistics shows these pathways have different properties, roles in cognition and temporal profiles. Human development also places revealing constraints on how children develop the ability to reason more abstractly as they grow up. We recognize language as a crucial cognitive faculty with several roles enabling the acquisition of abstract concepts indirectly. Three detailed case studies on body-specificity hypothesis, abstract verbs and mathematics are used to argue that a compelling case has accumulated in favour of the ultimate grounding of abstract concepts in an agent’s interaction with its world, primarily relying on the direct pathway. We consolidate the debate through multidisciplinary evidence for the idea that abstractness is a graded, rather than a binary property of concepts.

## 1. Introduction

We need concepts to organise our knowledge about the world. One aspect of these concepts is their abstractness, meaning the extent to which concepts reflect the idiosyncratic details of the experiences from which they are derived (Burgoon, Henderson & Markman, 2013). This establishes our experiences as the source for abstract concepts, but it leaves open the question of how exactly such concepts are formed. As a basis for the rest of the paper, we use a combination of the most commonly used definitions of abstraction to define it broadly as: a conceptual process by which general regularities and resulting concepts are derived from the usage and classification of specific examples (Barsalou et al., 2003a). The related notion of abstractness suggests that abstraction operates hierarchically, with some concepts being more abstract than others. The accepted view is that “democracy” does not just appear to be a more abstract concept than “pain”; instead, more abstract concepts are said to rely on cognitive processes that are in some important way different from those of more concrete concepts. The crucial open question about abstraction is what kinds of cognitive processes are the basis for how we understand more or less abstract concepts.

While many attempts have been made to explain abstraction, cognitive science lacks a consensus regarding the scope of these explanations (Yee, 2019). One set of examples are the embodied approaches, which argue, in one way or another, that cognition is non-trivially dependent upon the physical body (Wilson, 2002). Put another way, these approaches argue for different ways in which how we think and act depends on our bodies. Fincher-Kiefer (2019) summarises compelling empirical evidence for this idea from decades of research, centred around three major theories – metaphoric extensions (Lakoff & Johnson, 1980), action schemas (Glenberg & Robertson, 2000) and situated simulation (Barsalou, 2008). Besides compelling empirical support, embodied approaches also benefit from their consistency with the ascension of situated action in cognitive science, which is solving old problems by shifting emphasis from how humans think to how humans act within their environments (Robbins & Aydede, 2009). In this sense, embodied cognition reflects how cognitive science is maturing past the historically-privileged notions of mentalism, logic and a latent Cartesian dualism. Taken together, these reasons encourage making embodied approaches the starting point of a unifying look at abstract concepts.

Though the embodied paradigm offers compelling accounts of many of the basic features of cognition (Varela, Thompson & Rosch, 2017), it struggles with a full account of abstraction and abstract concepts specifically (Borghi et al., 2017). The challenge resides in the need to ground abstract concepts in concrete, sensorimotor interactions, or simulations (Barsalou, 1999). However, even within the embodied literature on abstraction, we are faced with a heterogeneous set of theoretical commitments. For instance, some attempts have adopted a representationalist view of abstract reasoning (Barsalou & Wiemer-Hastings, 2005), while others propose a radical embodied claim that completely eliminates the mental representations from its view of cognition (Thompson & Varela, 2001). That alone is the difference between retaining or completely abandoning one of the central concepts of cognitive science (Shea, 2018). This paper avoids getting bogged down in discussions of such underlying issues as much as possible. Instead, we use the considerable evidence for some role of embodiment in abstraction as a driver to approach the topic through the issue of symbol grounding more broadly (Harnad, 1990). That means we are addressing the question of how abstract concepts come to have meaning. To keep the discussion focussed, a large part of our paper presents three case studies through which we aim to breathe new life into different aspects of the theoretical debate.

One starting point from which to approach abstractions is the distinction that concrete concepts typically refer to categories of physical referents, whereas abstract concepts refer to sets of situations and events. This abstract–concrete distinction finds wide empirical support and dichotomization is a useful general approach in science when trying to contrast the two sets of entities. For example, Kousta et al. (2011) provide a number of factors by which to distinguish abstract and concrete concepts, such as imageability, context availability, or emotional valence. Aspects of these distinctions are also robustly supported by neuroimaging studies with patients with language disorders (Hoffman, 2016), as well as by findings indicating qualitatively distinct neural representational systems (Crutch & Warrington, 2005). Moreover, even studies that aim to move beyond the dichotomy report results that reinscribe a qualitative difference between the two types of concepts: abstract and concrete (Della Rosa et al., 2010). On the one hand, Wiener-Hastings & Xu (2010) identified differences between 18 abstract and 18 concrete words, when they asked participants to list features of different concepts: abstract/concrete concepts had more subjective/intrinsic properties respectively. On the other hand, based on ratings of 425 abstract concepts on 15 dimensions, Villani et al. (2019) concluded that the abstract–concrete dichotomy is too simple to properly understand differences between more concrete and more abstract concepts. In this paper, we reconcile such results by arguing that the differences these studies identify do not have to be interpreted in terms of a dichotomy between abstract and concrete. An alternative explanation is that there is a continuum of abstractness, from the most concrete to the most abstract concepts.

Alongside controversy around categories/degrees of abstractness, there is a different aspect of abstraction that cannot be agreed upon. Namely: how does the meaning of concepts, especially of abstract concepts, emerge in the first place? Put another way: how are abstract concepts grounded (Dove, 2011; Machery, 2009; Meteyard et al., 2012). This is the sense in which we are dealing with the symbol grounding problem (Harnad, 1990), which refers to how words – and symbols in general – first acquire their meaning. Abstract concepts, we suggest, can be thought of as just such a symbol (expressed by a word or a phrase), for which we must determine the source of its meaning. To understand the problem, imagine arriving in a new country in which all words and gestures are unfamiliar, and there is no way to begin to understand what someone is trying to communicate to you. The signs (words and gestures) have no meaning for you, because you cannot meaningfully relate them to anything you know. In other words, they are not grounded in your experiences of sensing/acting in the world, or your experiences of communicating with people using signs (a language, gestures) you do understand. Harnad’s (1990) point is that such experiences ground the meaning of all of our knowledge. Without grounding, we see someone mouthing and gesturing at us in a strange country, but understand nothing. That makes how concepts can be grounded a crucial question.

With the introduction of the concept of “grounding” it should be clarified that we use the term *embodied* in the context of the body, whereas *grounding* has a wider meaning, covering also other forms of interaction with the world (via language and social interactions). Grounding, not embodiment will be the central notion going forward, making the central question of this paper how abstract concepts are grounded. To reflect the distinction between embodiment specifically and grounding generally, we introduce the idea of two pathways of grounding. The first pathway captures embodiment in the sense of sensorimotor interactions with the world, interoception, or emotions. The second pathway builds on this to take into account how social aspects and language, in particular, add an additional route of grounding abstract concepts. This coincides with our idea of a continuum of abstractness that reflects different ways that concepts are grounded, specifically the extent to which they depend on either of the two pathways outlined above. This is consistent with differences between concrete and abstract concepts (e.g. Wiemer-Hastings & Xu, 2010) and differences between more or less abstract concepts as such (Villani et al, 2019, Villani et al, 2021a). We emphasise a continuum of abstractness mediated by two more/less direct pathways to make 1) gradedness and 2) directness of grounding the focal aspects of our contribution.

This paper is organised as follows. Section 2 is intended to shore up claims we have already made about the central role of embodiment, grounding and the role of two pathways. To do so, we briefly summarise the strong empirical support for embodiment of knowledge and outline the various roles language is assumed to play in cognition. This will provide grounds for developing our ideas in Section 3, on the basis of three detailed, purposefully chosen case studies of increasing abstractness. The case studies provide specific examples of different ways that concepts are grounded and provide further evidence for an embodied basis of abstraction. In Section 4, we complement the case studies with a review of neurodevelopmental literature that illustrates the contrasts and asymmetry between concrete and abstract domains. This provides both a closer look at the recent neuro-imaging data and a glance at how results from developmental studies shed light on a continuum of concreteness-abstractness. In Section 5, we summarise the supporting evidence for treating the abstract–concrete dichotomy as a continuum, and hence viewing abstractness as a graded phenomenon. Section 6 briefly summarises the major conclusions from the paper and offers a short outlook on open questions.

## 2. Embodied Cognition and Language

*Ahead of the main argument, this section outlines key aspects of an embodied approach to conceptual knowledge. Embodied cognition provides the basis for a continuum of abstractness by emphasising the role of interactions with our world across all domains of cognition. This claim is supported by a vast empirical literature that contains many internal disagreements about exactly how embodiment works. One particular challenge has been to provide an embodied account of language, which appears to provide a separate route to ground conceptual knowledge. Embodied approaches to abstraction argue that there are two pathways to grounding conceptual knowledge: one tied closely to sensing and acting in the world, and the other tied more to language and social interactions, which are themselves embodied in various ways*.

### 2.1 Embodied Approaches to Conceptual Knowledge

The starting point of this contribution is embodied cognition. In a nutshell, embodiment is one kind of grounding (Barsalou, 2010). Specifically, embodiment refers to the notion that conceptual knowledge derives its meaning from the apparatus, faculties and states of the (physical) body of the agent. For this, interactions with the environment are crucial (Barsalou, 2005), including those an agent has with itself, e.g. in interoception (Damasio, 1999).

The credited original description is that embodiment has two basic manifestations, one in the lived, experiential structure of the body, and the other in the body as the active vessel and immediate environment of cognition (Varela et al, 2017, Chapter 8). This reflects 1) an inner and 2) and outer view (Reid & Mgombelo, 2015). The corresponding claims are that 1) cognitive capacities depend on having a body with various sensorimotor capacities and 2) these capacities are embedded in the situation of the agent, encompassing biological, psychological and cultural factors (Varela et al. 2017, Chapter 8). This characterisation of embodiment reflects an enactivist or radically embodied perspective on embodiment that other authors, including ourselves, are not necessarily committed to or consistent with (Núñez, 1999).

From a neural perspective, that a certain cognitive ability is embodied is also understood as that ability being “mapped within our sensorimotor system” (Gallese & Lakoff, 2005, p. 3). In that sense, embodied knowledge is “structured by our constant encounter and interaction with the world via our bodies and brains” (Gallese & Lakoff, 2005, p. 3). As an example, consider grasping: the claim, from an embodied view, is that the information present in the sensorimotor system at the neural level is sufficient – and necessary – to create a conceptual structure of the concept “grasping/to grasp”. In this view, the sensorimotor system is necessary to understand a concept, because conceptual knowledge of the action “to grasp” is based on a sensorimotor simulation of grasping (Gallese & Lakoff, 2005). The simulation of grasping is the concept. Evidence for such a simulation comes from neuroscientific studies of humans either grasping or imagining grasping an object, which showed that both behaviours activate partially overlapping of the brain (Jeannerod, 1995; for a summary of neuroscientific data, see Jeannerod & Frak, 1999). Gallese & Lakoff (2005) go on that this same information is not only necessary, but also sufficient to ground conceptual knowledge, because all the sensorimotor structures through which we understand the world are present in the sensorimotor system, at least for concrete concepts. Staunch opposition to this view persists on the basis of data interpreted to show that even motor actions, arguably the most concrete concepts, are organised by conceptual, not sensorimotor properties (Bedny et al., 2008). The idea comes from functional magnetic resonance imaging (fMRI) data that show different patterns of reaction to verbs and nouns, which is taken to suggest that the partially-overlapping neural activity is rooted in event concepts (“I am moving a part of my body”) or grammatical information processed through these regions. In Bedny et al’s (2008) view, conceptual properties like these are of a higher order than “mere” sensorimotor cognition. This separates concepts from modal processing, i.e. information received through the sensory modalities. Doing so reiterates the question of how more abstract concepts may be grounded, if they are separate from the substrate of our sensory and motor experiences. What happens during the upstream integration of modal sensory processing that justifies talking about the kind of understanding in terms of conceptual properties that the abstract–concrete dichotomy depends on. The crux of this debate is more whether embodiment is necessary, and less whether it is sufficient to ground abstract concepts.

Whereas the following sections, and the case studies in particular, will address the controversy about whether or not we should think of concepts as being grounded, the take-away at this point is that embodied approaches focus on sensorimotor processing and the sensory modalities. In doing so, they emphasise that it is the ways that an agent perceives and the ways that it is able to act that determine what concepts and categories can be formed. Indeed, proponents go so far as to claim that the nature of concepts referred to by the very technical, linguistic notion of *aspect* – concepts denoting the structure of events and our reasoning about them – is such that they are ideally realised in premotor schemas (Gallese & Lakoff, 2005). This is a fine-grained argument from within cognitive linguistics that illustrates the external validity of embodied approaches by how its proponents have found neat overlaps and consistencies in the distinct concepts of sensorimotor and language cognition.

There exists a vast literature on such overlaps from the various theories of embodied cognition. These map cognitive linguistics onto embodied cognition, but also decision-making (Lepora & Pezzulo, 2015) or conceptual knowledge (Barsalou, 2008). However, this breadth incurs a variety of embodied theories that are not all completely compatible. One approach to taxonomizing these theories places different variants along a continuum, ranging from strongly embodied, through intermediate, to weakly embodied theories (Meteyard et al., 2012). The distinction between strong and weak embodiment is most closely related to the discussion whether embodied knowledge is sufficient to ground conceptual knowledge. Strong embodied theories argue yes, while weak theories accept that other types of knowledge are necessary as well. This distinction is important, because it captures one of the central issues of this article: to what extent abstract concepts are grounded via the first pathway.

Another distinction that is important is related to how different theories of embodied cognition can also be divided into groups based on the role that they assign to representations when it comes to concrete and abstract concepts (Scorolli et al., 2011). This distinction between theories that eliminate or include representations is more closely related to the question of whether grounding is necessary. Within the embodied approach, a frequent view is that embodiment is necessary to ground all conceptual knowledge, but that abstract and concrete concepts are represented differently in the two systems the perception and action system, and the language domain (e.g. Borghi et al., 2017). Based on recent neuro-imaging data, Pulvermüller (2018) argues that symbols are necessary to bind causal actions and their goals, from which they derive their meaning. The same study also attributes linguistic symbols (e.g. words) a key role in this binding when it comes to abstract concepts. This suggests that understanding abstract words may rely more on the linguistic system, whereas understanding concrete words depends more on the perception and action system. Besides reiterating our two pathway model, this also raises a crucial question about how exactly concepts (conceptual knowledge) and words (language) are related in the embodied approach.

### 2.2 Embodied Approaches to Language as a Second Pathway for Grounding

In general, many accounts on the organisation of semantic knowledge assume that the human cognitive system standing behind the acquisition of concepts consists of two main systems, the conceptual system and the linguistic system. Especially to those to whom concepts are inseparable from words, this is an indefeasible distinction. Nevertheless, separating the conceptual and linguistic systems has strong evolutionary support. At the same time that the human conceptual system appears more complex, in general, than that of other species, part of that complexity comes from the presence and importance of language in human lives and society (Barsalou, 2005; Šefránek, 2008; Evans, 2016). The cognitive processes within conceptual and linguistic systems also interact during development, also bootstrapping each other. On one hand, empirical evidence suggests that conceptual knowledge influences lexical acquisition in infancy (Booth, Waxman, & Huang, 2005). On the other hand, there is a lot of evidence in favour of the linguistic relativity hypothesis, according to which language shapes the way we think (e.g. Boroditsky, 2003). The crucial addition of our graded approach is to put these views on a continuum.

The two systems, conceptual and linguistic, are central to a variety of theoretical models of conceptual knowledge (for a recent overview see, e.g. Borghi, 2020). Within an embodied approach, the conceptual system is multimodal, meaning it is always tied to the senses and our ability to act. The main differences between such theories are in the assumed details (example theories include e.g. LASS by Barsalou et al., 2008; WAT by Borghi & Cimatti, 2009b; or LCCM by Evans, 2016). Most embodied approaches maintain that sensory modalities are the ultimate grounding of all conceptual knowledge, no matter how abstract. But other embodied approaches take the idea of distinct systems so far that they inadvertently reintroduce *amodal* concepts, concepts that are not tied to the body at all, in the end. Dove (2011), for example, offers the notion of *dis-embodied* concepts, positing a new kind of concepts, which “are dynamic and multimodal but, in contrast to other forms of embodied cognition, do not inherit semantic content from this embodiment [and are instead] embodied in the neurophysiological sense that they rely on sensorimotor simulation.” In Dove’s (2011) view, all linguistic representations of concepts are dis-embodied in this sense. He emphasises the value of language as an internalized, amodal symbol system itself grounded in an embodied substrate.^1^ The idea is reiterated in Dove (2018) where it is also suggested that language somehow acquires a fundamental character in our experience and thereby becomes its own fundamental grounding for concepts. We are concerned that this ignores that language mirrors the structure of sensorimotor interaction, rather than ever being independent of it. On the other hand, sensorimotor processes may not always be automatically activated during linguistic processing, since their activation may depend on the task and the context (see Tomasino & Rumiati, 2013 and references in that issue).

The challenge about language for embodied views of conceptual knowledge lies in determining how language plays an additional, but still embodied role for abstract concepts. For example, Borghi and Cimatti (2009b) specify an explicitly not-independent lexical semantic code, that allows conceptual content to be captured by relationships between different linguistic representations. To Borghi and Cimatti (2009b), in contrast to Dove (2011), language confers a number of new representational affordances through compositionality, recombination and a rich expressive medium (cf. Borghi & Binkofski, 2014). In their view, linguistic symbols cannot provide an amodal grounding for abstract concepts, as Dove (2011) suggests. Instead, as a secondary pathway (together with social interactions), they offer a particular cognitive scaffolding that exploits word associations to provide unique benefits to human cognition (cf. Clark, 2008).

Despite these disputes, there seems a general agreement about the fundamental importance of language in human cognition within embodied approaches, where a number of language roles have been identified: (1) Language allows linguistically-mediated communication (Evans, 2016), (2) it enables inner speech that helps us in monitoring our knowledge and in searching for meaning (Clark, 1998), (3) language is involved in controlling mental representations (Lupyan & Bergen, 2016), (4) it provides an efficient shortcut in certain tasks involving conceptual processing (Connell, 2019; Barsalou et al., 2008) and (5) it serves as a cognitive tool that enhances cognition as such in various ways and enables the acquisition and processing of abstract concepts (Mirolli & Parisi, 2011; Borghi, 2020), as discussed later. Consistently with the above roles, language also serves as an important medium for using conceptual metaphors. In these ways, language provides a second, indirect pathway for grounding conceptual knowledge.

## 3. Grounding Abstract Concepts

*We make the case that more and less abstract concepts depend on the two pathways of grounding in different ways. Less abstract (i.e. more concrete) concepts depend more directly on the body, while more abstract concepts are grounded in important ways by language and social interaction. Specific examples of concepts that are different in their degree of abstractness provide evidence for a continuum of abstractness, as more abstract concepts still depend on the body, but less directly so. At the very abstract end of the continuum, the two pathway hypothesis of grounding even provides an embodied basis of technical, mathematical concepts like “infinity”, which, in their actual form, cannot have a physical referent by definition*.

Despite the widely recognized important role of sensorimotor experience in grounding, especially in the context of concrete concepts, it has been recognized by many that an extension beyond a simple embodied approach is needed. Borghi et al. (2018) list three other forms of grounding, namely inner experience (interoception and metacognition), linguistic experience and social experience. Abstract concepts are all constituted differently and do not form a homogeneous set – numbers, emotions, evaluative concepts (e.g. aesthetic and moral ones) and social concepts are among examples of abstract concepts. This variety of abstract concepts is a challenge for any single explanation for how all of them are grounded.

Villani et al. (2021a) found evidence for this view in a study in which participants rated the difficulty of more concrete and more abstract concepts while also performing a second task. “Difficult” was not defined for the participants, but left to them to interpret. The tasks participants performed while they rated words were designed to interfere with conceptual processing in specific ways, in order to see if different concepts would be disrupted more by different tasks. Participants either 1) squeezed a ball in their hand, 2) chewed gum, 3) held the hand of a confederate or 4) held an instant warm/cold pack. The conditions were designed to interfere with 1) the hand motor system, 2) mouth motor system (related to speaking, therefore language), 3) social cognition and 4) interoception. The results showed that concepts vary widely in the extent to which these conditions affect difficulty ratings, compared to a control condition in which there was no second task to perform. More abstract concepts were rated as more difficult while holding an instant hot-cold pack, and concrete concepts appeared less affected by the mouth-motor system interference. This suggests abstract concepts might be more closely tied to interoception, while concrete concepts may depend less on language for their grounding. What the study also showed is that difficulty ratings of abstract concepts were affected differently by different conditions, such that some abstract concepts appeared to be interfered with much more than others by the manual versus the interoceptive task. This could be interpreted to mean that some abstract concepts rely more on direct grounding via the sensory-motor system, while others depend more on interoception or even mostly on indirect grounding via language. A second study by Villani et al. (2021b) not only reiterated that abstract concepts appear grounded in different ways from each other, but also showed that how concepts are grounded depends on personal experiences, in this case expertise in the legal domain. Their findings showed differences in how abstract concepts are understood by legal experts/non-experts, in terms of the ratings they gave words on 16 dimensions. These results showed that the more personal experience someone possessed with a concept, the more highly they rated it on direct dimensions like “hand and mouth involvement”. In general, this study reiterates the richness of abstract concepts and the differences between them in how they are grounded. For a complex example, the study found that emotion has a different significance for abstract concepts depending on expertise.

We tackle the problem that this richness of abstract concepts poses by grouping dimensions of grounding into two pathways. The first one is characterised by bodily experience, which includes both sensorimotor processes but also inner processes (interoception) – for a discussion of the role of interoception for abstract concepts see Connell, Lynott, and Banks (2018) – and affective states. The second pathway comes from interactions with others and includes both linguistic and social processes. Of course, language is very important for multiple reasons, as mentioned earlier, but it can only work if coupled by an earlier body-related mode of knowledge acquisition, in order to avoid the symbol grounding problem (see more about the developmental aspects in Section 4). The boundaries between the two pathways are not clear-cut, because, for example, social processes are often necessarily related to emotions (van Kleef et al., 2016). These can be analysed at individual, dyadic, group or cultural context but what seems to be important, though, are the properties of each concept that determine to what extent the concept is resistant to embodiment. In other words, from the perspective of the “owner” of an emotion, the same emotion can be evoked in different contexts which serve as its source of grounding. Experienced emotions are typically grounded through one’s own body, but they can also be grounded via observation within a social (not necessarily a linguistic) communication, potentially mediated by the mirror neuron system (Bekkali et al., 2021).

In the following, we provide evidence from three case studies, proceeding in an ascending order with respect to their “distance” from the “source” of embodiment. In the first case study, the body-specificity hypothesis points to a clear, immediate role of the body in the grounding of abstract concepts; the second case study discusses the grounding of abstract verbs, which we argue is often mediated through the body; as a third case study, we show how grounding mathematics reveals a highly abstract hierarchical set of concepts that poses a formidable challenge precisely because of how indirectly related to embodiment it appears. In fact, all three case studies illustrate the deep dependence of abstract concepts mostly on embodiment and partially on social interaction. In each case, the first pathway, via embodiment, provides the grounding and consequently constitutes the fundamental source of meaning. Whereas language, the second pathway, provides an additional, indirect means to ground especially abstract concepts. These case studies provide an opportunity to disentangle the multifaceted concept of abstraction one step at a time along a continuum of abstractness.

### 3.1 Body-Specificity Hypothesis (Case Study 1)

There is a range of evidence that motor experience shapes affective judgments, for example from an expert typist preferring easily typed letter pairs to a pair more difficult to type fluently (Van den Bergh, Vrana, & Eelen, 1990). Moreover, motor fluency has been implicated in a broad range of non-motor judgements (Oppenheimer, 2008). This case study focuses on a compelling series of five experiments makes the case that the association of valence (*good* vs. *bad*) with the left or right side of agent-centred space is the result of the differential experience of perceptuo-motor fluency with the corresponding hand (Casasanto, 2009). The five tasks, with five unique samples were 1) choosing where to draw a good/bad animal; 2) indicating where a corresponding animal should be drawn; 3) making an oral response about where animal should be drawn; 4) indicating preference for fictional creatures presented on left/right; 5) choosing, from a side-by-side presentation, which of two products to buy/which of two candidates to hire. In all five tasks – participants consistently associated positive valence with their dominant, and negative valence with their non-dominant hand (Casasanto, 2009). We interpret this as evidence that abstract concepts must, in some way, be tied to sensorimotor processes.

The body-specificity effect occurs in left-handed participants despite a strong cultural association of “right” with “good” (e.g. “my right-hand man”) and left with bad (e.g. “two left feet”). In the case of left-handers, the studies showed that the effect occurs not as a result of culturally-mediated metaphors but in opposition to them. Moreover, the assignment of right-is-good and left-is-bad is unsystematic, i.e. there is no idiom of “two right feet”, or one’s “left-hand woman”. In addition, differences on left/right ratings that were related to handedness were not present on top/bottom control conditions. A further study found positively and negatively-valenced speech acts to be preferentially associated with the dominant and non-dominant hand respectively (Casasanto & Jasmin, 2010). Beyond that, a subsequent study found that this association was reversed following unilateral stroke resulting in a change in motor fluency (Casasanto & Chrysikou, 2011). The same study observed a similar reversal for artificial handicaps induced in a laboratory setting after only minutes of experience. These results are strong evidence that motor experience plays a causal role in shaping abstract thought.

One way to explain this effect is via implicit motor simulations (Barsalou, 1999). This means that though the association is established and grounded in action and motor experience, the representation of the association may be decoupled from activity. Indeed, it is an open question to which extent, if at all, spatio-motor representations are constitutive of valenced concepts. Casasanto and colleagues fall back on the idea that it is impossible to demarcate concepts (cf. Wittgenstein & Anscombe, 1953), with the aim of using Wittgenstein’s concept of meaning-in-use to argue that the dissociable assignment of valenced labels in left- vs. right-handers implies that these groups have different concepts. It is not that handedness leads us to make different decisions, but to make them differently. By emphasising the act and process of decision making, Casasanto and colleagues shift the focus to what ultimately grounds the laterality of valence via perceptuo-motor fluency. In this view, concepts are not part of packages of preformed knowledge, but processes of activating stored information ad hoc based on sensorimotor experience (Casasanto & Lupyan, 2015). That way, this effect of how one uses one’s hands on how one thinks about and attributes abstract properties also makes a compelling case for embodiment (Casasanto, 2011b).

Complementing the behavioural data, an early question was whether the conventional hemispheric lateralization of valence in neuroimaging studies conducted with exclusively right-handed samples might be an artefact of body-specificity (Casasanto, 2009), especially considering functional lateralization as a result of handedness (Casasanto, Hagoort, & Willems, 2009). There is already evidence of lateralized activity for motor imagery of hand-related action and for reading hand-action related phrases in premotor cortex (PMC) (Willems et al., 2009). Moreover, the application of disruptive transcranial magnetic stimulation (TMS) reiterated that premotor activity was functional, not epiphenomenal to hand-related language processing (Willems et al., 2011). Specifically, bursts of TMS over lateral PMC reduced reading rates. Taken together, this points to an embodied semantics in the neural processing of one set of abstract concepts, namely valence.

Given the role that the body plays in emotion (Damasio, 1999), and the body-specificity of valenced, often emotionally-laden concepts, there is a case to be made that body-specific patterns of activation mediate a functional connection between the neural substrates of motor control and emotional mechanisms such as motivation, specifically approach-avoidance systems (Casasanto, 2011a). Indeed, there is evidence of neurophysiological correlates of handedness across the human frontal lobe (Brookshire & Casasanto, 2012), which can be interpreted as evidence of such a functional link. This converges with data that support the differential grounding of abstract concepts in emotional states in contrast to more concrete concepts (Kousta et al., 2011). Through this link to emotion and its neural correlates, the body-specificity hypothesis provides evidence that motor fluency, a fundamentally embodied experience, is one of the organising factors of conceptual structure. Casasanto and colleagues tie the grounding of valence to embodiment directly. These studies attribute a subordinate role to the second pathway of grounding, namely social interactions and culture transmission. The next case study moves on to consider a different way that our experience ground abstract concepts.

### 3.2 Conceptual Metaphors of Abstract Verbs (Case Study 2)

Different word classes appear to be processed by partially dissociable neural circuits (Wiemer-Hastings & Xu, 2005). Verbs, in particular, can also be characterised in terms of their relative embodiment – a measure derived from Borghi and Cimatti (2009a) that captures the bodily sense and strength thereof with which a given verb is associated (Sidhu et al., 2014). On three different tasks, Sidhu et al. (2014) showed a facilitative effect of relative embodiment in terms of reduced reaction times, even when controlling for lexical factors such as imagability. From these results, embodiment appears to be a central aspect of verb processing and by extension of verb meaning.

Previous work suggests there are also differences within the verb class (Barsalou & Wiemer- Hastings, 2005). For example, neuroimaging data show that, in terms of their involvement of the cortical motor system, abstract verbs with motor stems (e.g. German *begreifen*, “to *grasp* an idea”) are dissociable from verbs with explicit motor meanings (e.g. German *greifen*, “to *grasp* a cup”), but not distinguishable from verbs with abstract stems (Rüschemeyer, Brass & Friederici, 2007). At first, this suggests that having a motor stem rather than an abstract stem does not make verbs more connected to the cortical motor system. However, this does not necessarily weigh against an embodiment of motor-stem abstract verbs. It establishes a similar processing network for abstract verbs, whether they possess a motor stem or not. This could reflect the equal embodiment of abstract verbs irrespective of their morphological form.

Both abstract and concrete verbs extensively, but differentially recruit sensorimotor cortices (Sakreida et al., 2013). Both kinds of verbs appear embodied to some extent, but rely on the sensorimotor and conceptual linguistic subsystems to different extents. This is in line with various attempts at synthesis (Dove, 2011; Arbib, 2017) and with our idea of a continuum of abstractness. One interesting study showed that the processing of abstract actions recruits motor systems independent of the presentation format (schematic visual displays versus words) (Quandt, Lee & Chatterjee, 2017). This indicates that there is some level of processing beyond the modality in which we usually experience an action. However, the activity appears specific to individual abstract actions, without a clear pattern. As a result, this section will examine an individual abstract verb to better understand how it is grounded, and thereby to improve our understanding of the grounding of this word class as a whole.

One example is the intransitive verb “to meditate”, which means “to engage in mental exercise (such as concentration on one’s breathing or repetition of a mantra) for the purpose of reaching a heightened level of spiritual awareness” (Merriam-Webster, 2016). At first this seems like a concrete verb, but a closer look at the goal-directed nature reveals this may not be the case. This becomes more apparent in the definition by editors of the American Heritage Dictionaries (2018) of “to meditate” as “to train, calm, or empty the mind, often by achieving an altered state, as by focusing on a single object, especially as a form of religious practice in Buddhism or Hinduism.” That definition is not in terms of any specific actions, so much as in terms of the purpose of this action, which is the abstract emptying or calming of the mind. As an abstract verb, “to meditate” is a particularly lucid example of how each concept is grounded through both pathways, which also makes it a good example of the gradedness of abstractness–concreteness. As a verb, it refers to an action, from which the concept draws a certain concreteness. At the same time, the theory of meditation contributes a rich abstract character that explicitly embeds the act of meditating in a system of values and norms. How we understand the concept falls somewhere in between.

One way to approach abstract verbs like this is by taking into consideration the two types of knowledge under discussion: conceptual/intellectual and embodied. This reflects a different aspect of differences in how directly concepts are acquired, as in how immediate our experience of their referent is. Starting from the experiential nature of some knowledge, Pagis (2010) explored the concept of dissatisfaction in Buddhist tradition to show that individuals who ‘possess’ an abstract concept prior to lived experience, have a rather vague notion of what that concept represents. They can connect to it “intellectually”, but it often hardly poses any meaning to them. Through repeated acts of lived experience that constitute the referent of the concept, participants reported that they acquired a new meaning for the concept. This example also reflects the fundamental role of non-sensorimotor sources of knowledge in the grounded paradigm, such as meta-cognition and affect (Barsalou, 2016). At the same time, the example outlines an experiential understanding that is rooted in the sensory impressions and actions that make up that experience. However, this leaves open to what extent the representation of the concept of dissatisfaction changes as someone becomes more familiar with it in an experiential way.

To understand how the basic representation of “to meditate” first develops, it is worth noting that the definitions cited above make use of embodied language, like the image of “emptying (pouring out) a mind”, or “training the mind (like a muscle)”. These kinds of images are the subject of the theory of conceptual metaphors, one of the first embodied accounts of abstract thought (Lakoff & Johnson, 1980). In this view, metaphors shape our conceptual system, which in turn, shapes our view of the world.^2^ This results not only in metaphorical speech, but also in metaphorical thought, where the latter precedes the former. Within conceptual metaphor theory, understanding a grasping movement is taken to facilitate the comprehension of metaphorical phrases like “grasping a concept” (Gibbs, 2011). Or the act of balancing our bodies when we move in the world, for example, can serve as the embodied metaphoric foundation for the concept of justice, which is often imagined as a scale to be balanced (Antle, Corness & Bevans, 2011). In this view, abstract concepts are grounded by mapping our experiences of balancing our bodies onto the concept of justice. Through this mapping, we understand that justice has something to do with managing excess and deficiency. At the same time, the converse need not hold. So deficits in the ability to balance need not necessarily produce difficulties understanding the concept of justice – though a possible relationship, especially during childhood, offers an interesting avenue of research.

The question of the mechanism of this mapping remains. In conceptual metaphor theory, it is our experiences of the co-occurrence of a source (balancing) and a target (justice) that creates a neural mapping to connect the feeling of balancing with the concept of justice (Gallese & Lakoff, 2005). The circuit that is formed in this way is the metaphor and the experience results in the metaphor of justice-is-balance in the cognitive domain (Lakoff, 2014). In this model, since the concrete experience of balancing involves the sensorimotor system, that system is indirectly involved in representing abstract concepts, like justice, that are based on metaphor of balance. In this account, the meaning of those concepts depends on being mapped back into the concrete domain, and therefore back to sensorimotor representations. Pulvermüller (2018) investigated the mapping that conceptual metaphor theory proposes at the neuro-mechanistic level of distributed cortical circuits for the abstract verb “to cause”. He argues that abstract concepts and words can be learned by and grounded through real-life interaction, because it is only through the neural activity associated with our experiences that the meaning of abstract concepts can be consolidated.

It is important to keep a distinction in mind between concepts and words. On a neural level, associations between concepts are not straightforward, because concepts form dense multimodal networks, related via mental simulators (Barsalou et al. 2008). By contrast, associations between words are more direct since lexical units are much simpler entities. This allows the formation of fast associative lexical networks. To some extent, this reflects the double structure of the two grounding pathways again. In fact, our two-pathway model recapitulates an aspect of conceptual metaphor theory when we distinguish between a “deeper” understanding which has a motor component (within the first grounding pathway), and “shallower” understanding that does not have it (second grounding pathway). This distinction was originally proposed within the direct matching hypothesis (Rizzolatti & Sinigaglia, 2010), which defines deeper understanding as being mediated by mapping observed actions to one’s own motor repertoire via mirror neurons (here, the term “direct” is unrelated to its use in the context of pathways). Consequently, shallower understanding results from cases where no such mapping is available (e.g. observing someone doing a somersault without his/her own experience). By extending this idea to experiential context, it is still possible to conceptually understand something (e.g. justice; via language and social interactions), without an understanding that maps the concept directly to embodied experience. In this account, an embodied, first pathway route just provides an added value, understanding “from inside”. That is not to say that a second pathway grounding is not also embodied, only it is embodied indirectly, via the way that language and social interactions themselves are ultimately grounded in embodiment. Within conceptual metaphor theory, this distinction produces the “dimensions” that constitute the representation of the concept. The motor dimension is one of these dimensions and its absence does not eliminate understanding as such. Such a distinction will be useful also in other contexts throughout the paper.^3^ The next case study takes up this point and moves even further along the continuum of abstractness, to concepts that cannot have a physical referent by definition. This would appear to rule out any direct mapping at all to the sensorimotor system, suggesting they are entirely dependent on the second pathway.

### 3.3 Embodying Maths (Case Study 3)

On the surface, it appears that mathematical knowledge should pose a formidable challenge to embodied cognition. Matrices, vector-spaces and imaginary numbers are all conceptually-rich entities that do not immediately suggest a practical, action-based origin. Of course, the foregoing discussion has opened the possibility of grounding concepts in sensorimotor experience indirectly, via simulation or conceptual metaphors. Mathematics education reveals that we intuitively exploit the scaffolding role of physical engagement during the acquisition of mathematical concepts – just consider the role of counting on fingers during early numeracy training (Hutto, Kirchhoff & Abrahamson, 2015).

This example lends itself to an interpretation according to which mathematical cognition is based on interactive dynamics with limited to no involvement of contents or concepts – consistent even with the radical embodied and enactive views (Chemero, 2011). Hutto, Kirchhoff & Myin (2014) propose a view partially based on basic numerical capacities that exist long before the formation of mathematical concepts in human development. In part, the argument also rests on a phylogenetic perspective that emphasises the role of an environment that affords dynamic interaction as the structuring force behind human cognitive abilities. Again, it is our experience with them that accounts for the physical reality that abstract concepts, in this case mathematical ones, appear to have for us; the key may be our basic experiences of size, weight and even emotive significance (Núñez, 1997).

Previous work has argued that an embodied understanding of number concepts can account for heuristics and biases in mental arithmetic (Fischer & Shaki, 2018). Hayes and Kraemer (2017) also discuss the grounding provided through laboratory-based and hands-on methodologies in science and engineering, but somewhat neglect mathematics, which we rectify here. To do so, we focus on the Kinemathics project (Abrahamson et al 2011, 2012; Abrahamson & Trninic, 2015), as summarised in Hutto et al (2015). The project was intended to design a tutoring method to help students (United States’ grades 4–6) understand the concept of proportional relations. The solution that was developed encapsulates the role of embodied learning in mathematics. In the implementation of the method analysed for the Kinemathics project, Howison et al (2011) invited students to sit at a desk in front of a large screen. Students see that the height at which they position their hands in front of a screen moves two cursors up and down, one for each hand. They are then given the instruction to “make the screen green” by moving their hands in front of the screen. To make the screen green, students have to hold their hands apart according to a fixed interval that is unknown to them. The next instruction they receive is to keep the screen green while moving their hands. This provides an embodied basis to understand proportionality. The task is successively modified as students learn to orient and move their hands according to different ratios. First a grid is introduced in the background, then the gridlines are numbered, then students start moving the cursors by inputting numbers on a keyboard. This process gradually adds layers of abstraction on top of the newly learned manual skill to slowly introduce mathematical concepts and notation that are grounded in sensorimotor interaction.

Micro-ethnographic studies of human development suggest that, throughout cognition, it is likely goal-oriented physical interaction such as this that acts as the psychological basis of the human ability to recognize and reproduce symbolic tokens (Hutto et al, 2015; cf. Piaget, Inhelder, & Szeminska, 1960). In a similar sense, physical operations on objects may constitute the basis of arithmetic operations and their signs, which simply re-encode a previously implicit understanding of the corresponding transformation (Radford, 2013). A child might play with two groups of objects, combine them into a single group being aware of the persistence of the total, and only later acquire the arithmetic notion of addition and the corresponding notation. This is what is reported in the Kinemathics project. Here, the grounded cognition literature happens upon what seems to be a basic process of knowledge acquisition during child development, which leads it to embrace nativist constraints on how we are able to learn as children (Barsalou, 2016). The idea is that pre-symbolic knowledge identified in sensorimotor interactions captures a precursor of mathematical knowledge.

What the Kinemathics studies suggest is that the episodes with the training method constitute the grounding of both the simple mathematical concepts they refer to and, separately, the grounding of highly abstract mathematical notions. To substantiate this, a subsequent study used non-invasive eye-tracking to study patterns in the children’s eye gaze (Hutto et al, 2015). These data indicate that immediately prior to learning a new ratio between their hands, children’s eye-gaze was directed towards the relevant position that marked the target ratio (e,g, half way up one side for a ratio of 1:2). Then students typically exclaimed they had understood the task. This shows an attentional and motor understanding prior to any knowledge of having “got it”.

One concept that is important for understanding these studies, and by extension for understanding the embodied argument presented here, is the *attentional anchor* (Hutto et al, 2015). The term refers to the focus of an agent’s attention during a specific interaction with its environment. The anchor is made explicit to the learner as her skill increases by engaging in related tasks (Ingold, 2000). This anchor may serve as a higher-order invariant, rooted in situated motor interaction that grounds action in order to reduce complexity and enhance control (Kostrubiec et al., 2012). Achieving attentional anchors is positively related to performance and learning rate, while the anchor itself reflects the convergence of different idiosyncratic solutions (Hutto et al, 2015). In the Kinemathics studies, the physical grounding offered by the interval between the participants’ hands attracted their attention and was brought forward in experience, as an entity to be manipulated and monitored (Hutto et al, 2015). Individuals adjusted their motor behaviour prior to their own awareness of such changes (Kelso, 1984), reflecting what may be a preferred processing route for conceptual knowledge through physical interaction, as well as a facilitating role of the sensorimotor system for understanding abstract concepts. The interval that acts as an attentional anchor is brought into existence by exploratory motor behaviour and becomes a stimulus for learning in its own right (Hutto et al., 2015). Furthermore, that gaze preceded speech about a relevant location during the task, as revealed by the eye-tracking data, suggests that it and the learned motor schemas may be what provides the basis for discriminations made in speech later on, via implicit, sensorimotor based reasoning. Consistent with the role this suggests that sensorimotor interactions have for learning, Abrahamson & Trninic (2015) reported a positive learning outcome for students who engaged in manual exercises directly or vicariously.

Evidence from adults substantiates this claim by linking gesturing with how (well) we understand mathematical concepts. On the one hand, adults use embodied language in the domain of mathematics. For example, mathematicians refer to “never *going* beyond a limit”. Like with conceptual metaphors though, the question remains whether this aspect of language points to anything like a functional embodiment of our understanding of the underlying concepts. The notions employed, e.g.“*approaching* a limit”, are likely rooted in physical experience. Without a distinct mathematical coding, this at least suggests that mathematics recruits embodied notions similar to how conceptual metaphors work (Marghetis & Núñez, 2013). It also suggests that mathematical knowledge builds on domain-general cognitive mechanisms, as would be expected (Fauconnier & Turner, 2003).

On the other hand, gesturing has been shown to be functional in language (Pulvermüller et al., 2005). Gesturing also facilitates the acquisition of new concepts in proportion to the faithfulness of gestures to the mathematical notion they are related to (Goldin-Meadow, Cook, & Mitchell, 2009). By itself, that gestures are used to communicate meaning does not necessarily implicate embodiment in the fundamental encoding of an abstract concept being communicated through gestures. However, co-speech gesturing also reflects implicit cognitive processes not evident in speech (Goodwin, 2000). Marghetis and Núñez (2013) argue that such implicit, embodied processes are why their data show that dynamic thought, which is reflected in increased gesture dynamism, facilitated the generation of rigorous mathematical proofs. Taken alone, this still does not disambiguate the role of gesture dynamism however, ie. whether it structures mathematical thought or simply supports how we understand or communicate mathematical knowledge. Nonetheless, Zdrazilova, Sidhu & Pexman (2018) reiterate a crucial role for gestures in communicating and grounding abstract meaning.

Crucial evidence comes from the ethnographic side again, as it did with Kinemathics. One study noted that gesturing while explaining scientific figures mirrors the physical structure of the recording device used to collect the represented data (Roth & Lawless, 2002). That puts embodiment forward as the basic means of understanding and/or communicating concepts. In the context of mathematics, gesturing is even, and unusually so, recruited in association with abstract noun phrases, like “limit” (McNeill, 1992), despite being preferentially related to verbs in other contexts. Recalling that language may be built upon the gesturing system on the evolutionary scale (Arbib, 2017), these studies suggest that the gesturing practice of mathematics educators does provide a meaningful source of grounding during the pedagogical interaction, for for themselves and, by enacted example, for their students (Roth & Lawless, 2002). Reminiscent of Barsalou et al. (2003b), this also emphasises the social interaction at the root of learning (Cobb, 1994; Voigt, 2013) – see also Balacheff (1991).

All of this is evidence for an involvement of sensorimotor interaction in learning and doing mathematics. However, it has been difficult to show that this embodied knowledge constitutes the ultimate grounding of mathematical concepts. Practically acquiring a mathematical concept remains a very different process from acquiring a manual skill like playing piano or basketball (Hutto et al, 2015). Indeed, embodied interaction with the display used to teach proportion in Kinemathics still requires rich mental constructions (de Freitas & Sinclair, 2012), which may be of a different kind than those that subserve the practice of piano or basketball. On the other hand, gesture itself has been argued to reflect the underlying perceptual and motor simulations that constitute the sediment of embodied language and cognition generally (Hostetter & Alibali, 2008). In this case, the functional act of gesturing would indicate the deep embodiment of the multimodal concept in reference to which it occurs.

What all of this is distinct evidence for then, is that mathematical concepts can be brought forth through action (Abrahamson & Trninic, 2015). It also shows that this interaction can be conceptualised in dynamical terms (Hutto et al., 2015). It also seems clear that embodied engagement has advantages beyond strictly abstract learning methods (Marghetis & Núñez, 2013). To that extent, it is even exhibited by adult experts (Roth & Lawless, 2002). Taken together, this is compelling evidence from various sources that sensorimotor interaction is one fundamental mode of cognitive operation with respect to mathematics. Against such a backdrop, it may be up to the disembodied paradigm to substantiate the long undisputed claim that mathematical knowledge is an encapsulated, purely abstract domain (Reid & Mgombelo, 2015).

### 3.4 Other abstract concepts

Obviously, there exist even more abstract concepts than the examples mentioned above. Nevertheless, we think that our explanatory approach would be the same. It seems true that the more abstract the concept is, the more reliance on the second pathway it requires. In the language pathway, the contextual words should provide information about the semantics of the target word. Regarding grammatical categories, it is difficult to say what can be more abstract, whether nouns or verbs. Even though there exist large collections of word concreteness ratings for various languages (e.g. Brysbaert, Warriner & Kuperman, 2014, for English), their distributions of ratings are highly overlapping and have very large variance. But, regardless of the target grammatical category, recent interesting results by Naumann, Frassinelli and Schulte im Walde (2018) reveal that 1) concrete target nouns, verbs and adjectives primarily co-occur with concrete nouns, but *abstract* verbs and adjectives, while 2) abstract target words (nouns, verbs, adjectives) primarily co-occur with *abstract* words. This means that the significant portion of both concrete and abstract target word context refers to words that themselves are difficult to ground, pointing to the symbolic merry-go-round (Harnad, 1990). On this semiotic carousel ride, we keep spinning in circles so long as we try to ground abstract concepts on other abstract concepts. The only way off the merry-go-round is to find concrete concepts in which to ground more abstract ones. With regard to Naumann et al (2018) we predict that only the complementary portion of contexts provided by concrete words mediates second pathway grounding through language. In our three case studies, we focus on the contribution of the first pathway, which serves as the primary source of grounding. However the “strength” of that direct grounding may be, admittedly, negatively correlated with the abstractness of the concept.

A crucial aspect that will need to be examined more closely is how exactly learning might proceed from concrete to more embodied abstract (first pathway) and finally more socially/interactively mediated abstract concepts (second pathway). The acquisition of mathematical knowledge, for example, is a gradual process that has to proceed in a certain, incremental manner as we grow up. To address this, the next section outlines a neurodevelopmental perspective that provides another fruitful source of evidence for the dynamics of abstract cognition that can be integrated into the overall picture.

## 4. Neurodevelopmental Perspective on Abstract Concepts

*Looking at how our ability to understand concepts develops through childhood is another way to approach degrees of abstractness. Various neurodevelopmental studies have shown that abstract conceptual knowledge builds on understanding concrete concepts in particular and reliable ways. This supports an embodied view of abstraction, if it can be shown that the concrete basis continues to underpin how we understand abstract concepts, even once children develop the ability to reason more abstractly. A neurodevelopmental view also sheds light on how the grounding of abstract concepts shifts from being embodied (first pathway) to being more strongly tied to language and social interaction (second pathway)*.

Delving into the neurodevelopmental perspective offers great and essential insight into how the ability to think abstractly arises (Yee, 2019). As argued by Barsalou, Dutriaux and Scheepers (2018), abstract thinking is the ability to integrate various relational properties of elements in a given situation. Humans tend to fully acquire this ability only in late adolescence. Evidence suggests that growth of new connections and myelination of neurons lasts until early adulthood (Nickel & Gu, 2018). Recent studies have shown that one of the most prominent parts of the brain responsible for relational/abstract thinking is the rostro lateral prefrontal cortex (RLPFC) (Wendelken et al., 2011; Wright et al., 2008; Dumontheil, 2014). RLPFC has a prolonged development in children. It has been shown that this neural structure plays a vital role in processing relational integration of stimuli and aspects of episodic memory retrieval (Dumontheil, 2014). During tests of processing semantic relations, RLPFC had engaged later in children than in adults. In children it was activated after a decision was made and the older the child was, the faster the activation and the bigger the role it played in correctly finishing the task (Wright et al., 2008). This neuroanatomical evidence proves what is readily observable in children, they tend to acquire concrete concepts much faster than abstract concepts. Although our ability to think abstractly comes only later in life, it seems that it is with us from the start.

Concept acquisition starts with natural categories and as mentioned above, goes roughly from concrete to abstract. Nevertheless, when it comes to common categories that are hierarchically organised, the acquisition starts at the basic level (Rosch, 1973) that presumably provides (ecologically) the most relevant conceptual information and/or right amount of variability among instances (e.g. dog, chair). Subordinate level categories are characterised by a low degree of generality and by clearly identifiable, detailed and specific features (e.g. Dalmatian, a rocking chair). On the contrary, superordinate level typically implies a high degree of generality and allows to store general information (e.g. mammal, furniture). Hence, subordinate categories are more concrete than basic categories, which in turn are more concrete than superordinate categories. Interestingly, however, despite the order of increasing abstractness, the acquisition of knowledge in children does not in this case proceed bottom up (Bloom, 2002).

Actually, according to Borghi and Binkofski (2014), this type of abstraction (e.g. mammal vs. dog) should be distinguished from abstractness that does not, unlike the former, provide concrete, perceivable referents (e.g. an electron). They contend that abstractness, not abstraction, is the real problem posed by abstract concepts. We view abstraction and abstractness as related but distinct problems posed by abstract concepts. In a developmental context, we can also return to numbers. Izard et al. (2009) identify a fundamental prerequisite for abstraction, namely the ability to match events to one another, which they show is already present in newborns. They conclude that some abstract numerical representations are present from birth. Arguably therefore, this ability should also be present in utero, and raises a question of when and how exactly it develops. Such findings raise the profile of nativist approaches and indicate that a number of essential functions formed prior to birth. Language adds an additional layer of complexity that most likely builds on such a fundamental function, but also requires abilities only acquired during post-natal experience. Developmentally speaking, abstraction then remains difficult, for example, because using some superordinate labels is itself a challenge, among other reasons due to the sheer variability of referents (including those that very much depart from typical examples; e.g. a whale does not really look like a mammal). Acquiring this skill appears to take time.

The common developmental trajectory also points to some differences between more concrete and more abstract concepts; it is not by chance that more abstract concepts can only be acquired after many more concrete concepts have been learned and labelled (by words). Referential uncertainty of abstract words may be the reason why they are acquired later than concrete words (Bergelson & Swingley, 2013). In more detail, one can expect that the age of acquisition of a concept linked to a word will be correlated with its degree of abstractness. This scaffolding effect is consistent with a view on development that argues that infants exploit what is offered by their environment and their own bodies to construct an understanding of their world. Such a view introduces a distinction between objects that can be manipulated and ones that cannot. Indeed, Scorolli et al. (2011) argue that differences between more abstract and more concrete concepts may be traceable to the way they are acquired. They suggest that abstract concepts are acquired more linguistically than perceptually, and that this mode of acquisition produces the way they remain grounded, also in neural terms. On the other hand, despite the crucial role of language for acquiring abstract concepts, it seems that from neural perspective, it is not lexical-grammatical categories that drive the organisation of abstract concepts in the brain but their semantic properties (Moseley & Pulvermüller, 2014).

D’Angiulli, Griffiths, and Marmolejo-Ramos (2015) develop a broad framework of the grounding of conceptual knowledge of preschool children, using a comprehensive Event-Related Potential (ERP) investigation of a picture matching task following aural presentation of more abstract or more concrete words. Their approach draws on results consistent with the dynamic interaction vision–language approach (DIVLA) (Mishra & Marmolejo-Ramos, 2010). While this approach is an embodied one, it differs from the mainstream by emphasising the visual aspects of sensorimotor interactions, and suggests these have primacy over motor components. While other modalities also contribute to linguistic processing (e.g. audition, especially considering the social role of prosody), evidence of a central role of visual processing suggests this is an especially important system, with a special role in the phylo- and ontogenic development of language (Givón, 2002).

The results of Mishra and Marmolejo-Ramos (2010) can be interpreted as evidence of a weak, but pervasive embodiment. The ERP-evidence revealed a dominant presence of activity patterns associated with visual perceptual processing. This was the case both for more concrete and more abstract concepts. Concepts on different ends of the continuum tended to differ on the amount of time during which they engaged visual processing, suggesting a persistent involvement of the vision-language interface for abstract words. ERPs also offer independent indications of the involvement of linguistic processes. The overall ERP dynamics suggests a fast visualisation for more concrete concepts, in contrast with a gradual embodiment of more abstract concepts, followed by visual processing and a broad recruitment of the vision-language interface for the purpose of word comprehension. This can be seen as a consequence of the mode of acquisition, work on which also suggests a developmental trajectory of conceptual knowledge in which more abstract concepts require more and more complex visualisations (Borghi & Binkofski, 2014). The particular role of social components of more abstract concepts is a prime example of the additional complexity that takes a bigger role for more abstract concepts. In the context of DIVLA, it should be made clear that the perceptual aspects of sensorimotor interactions contribute at least equally, and possibly more than motor components (Mishra & Marmolejo-Ramos, 2010).

These results are discussed here because they offer an account of cognitive processes necessary in the developmental context. Importantly, visual and other modal processes take such an essential role regardless of whether a concept is more abstract or more concrete. Reflecting on the major developmental contribution of visual contexts of word learning, Iossifova and Marmolejo-Ramos (2013) found evidence of shift towards body-based processing of abstract concepts for blind children, compared to sighted and visuomotorily impaired children. This also reiterates the central role of sensorimotor aspects of grounding. Further underwriting this for the developing brain, it appears that sensory input during action perception only activates the motor system after experience of own interactions with objects (James & Swain, 2011). In the context of the developing sensorimotor system and sensorimotor representations, this places emphasis on one’s own actions (motor and sensory domain), rather than perceived actions (sensory domain). Own actions are motor and sensory since there is online sensory perception of own actions, also via proprioception. That being the case, an early function of motor association areas may be the association of previous experiences and current sensory input.

Importantly, recalling the indirect pathway of grounding referred to by the present work, there is also a social dimension to DIVLA; this highlights the role of the shared construction of social situations through aspects including gestures and prosody, which play a central role not just in mature language, but also in the process of language acquisition and cognitive development in general, which is in itself fundamentally based on sensorimotor interactions (Smith, 2005; Vygotsky, 1964). In this context, words can be understood as tools (Borghi, 2020), and it does appear that linguistic labels, in addition to and built on sensorimotor experience, are crucial to the developmental acquisition both of language and of conceptual knowledge. For example, the higher age at which children tend to acquire abstract concepts might be accounted for by the referential uncertainty that arises because the physical referent of an abstract is word is absent more often during mother-infant speech, than is the case for concrete words (Bergelson & Swingley, 2013). Crucially, this points to infants’ social-cognitive abilities as important aspects of concept acquisition.

Concerning social interactions, these are believed to facilitate the acquisition of more abstract concepts, for instance, mediated by valence. Given the profound relevance of emotion to human life, it is not unsurprising that both positive and negative more abstract words are acquired earlier than neutral ones (Ponari, Norbury & Vigliocco, 2016). Nonetheless, this points to another important driver of the development of conceptual knowledge. Meanwhile, valence is only one aspect of a dimensional approach to emotions and similar effects may exist for other affective factors, e.g. arousal. The age of acquisition rating method used in this study is broadly validated (Scorolli et al., 2011). This method has revealed a central role of the abstractness–concreteness of the noun during language acquisition, which may facilitate the acquisition of more abstract verbs over that of similarly abstract nouns. Consistent with this linguistic contextual effect, Della Rosa et al. (2010) offer the mode of acquisition (Wauters et al., 2003) as an independent predictor of abstractness–concreteness, in line with the different contribution of perceptual, motor and linguistic processes during development. To these, the affective processes should also be added.

Recalling the mapping of abstractness–concreteness and directness of grounding presented at the outset of the section, an intuitive hypothesis is that the more abstract the word is, the more difficult it is to acquire (manifested by its later age of acquisition). However, this does not hold along the entire continuum, as we have seen in case of hierarchically organised object categories.

## 5. Graded Abstractness

*We have stressed three points about abstractness. First, conceptual knowledge is fundamentally grounded, and dependent upon embodied experience in interaction with the world. Second, the production of abstract concepts is not qualitatively different from that of concrete concepts; as such, they differ only quantitatively in the extent to which they depend on certain information and not, in principle, in the kinds of information (sources of grounding) a given concept may depend upon. Third, given the nature of conceptual knowledge and the absence of a qualitative distinction of concrete and abstract concepts, the abstractness–concreteness dimension should be thought of as a continuum, as argued also by others*.

This paper has outlined several different processes whereby grounded knowledge is produced, some referring more, some less directly to the sensory periphery. This being the case, we suggest that these two scales – abstractness–concreteness and directness of grounding – can be mapped onto one another. Then, the longer and more complex the route back through metaphors, schemata and simulations, the more abstract a concept will appear. But, there is a risk that this mapping only reflects the difficulty of accounting for different abstract concepts in embodied terms; for whatever reason, the grounding of a particular concept may be especially elusive and therefore tempt us to conclude this must be a very abstract concept. The challenge is to find a principled solution.

The situation is that the degree of abstractness seems correlated with the “distance” to – and maybe the complexity of – the sensorimotor processes. This relates to the work on the BOI effect, which measures how “easy to interact with” words’ referents are perceived. Past research revealed facilitated lexical and semantic processing for words rated high in BOI than for words rated low in BOI (Hargreaves et al., 2012). Unsurprisingly then, there are close similarities between objects high in BOI and the referents of concrete words, and likewise for low BOI and those of abstract verbs. This close relation of difficulty (of imagining interacting with something) and concreteness-abstractness reinforces the idea that more abstract concepts are further from their sensorimotor grounding. For abstract concepts, this “distance” may be much larger because their meaning is mediated by related words (hence involving the linguistic system), that in turn might have a shorter distance to sensorimotor grounding. However, in our view, this does not justify positing the concept of hybrid grounding (e.g. Dove, 2011; Louwerse, 2011). Words as contextual symbols cannot often provide grounding themselves since they are primarily abstract (Naumann, Frassinelli & Schulte im Walde, 2018). Still, the second pathway also provides concrete context consisting of concrete words that provide room for direct grounding. In the paper, we emphasise the singularly fundamental role of embodiment, which persists as the primary grounding pathway throughout complex linguistic interactions that are necessarily built upon it. And as mentioned at the end of Section 2, language has multiple important roles in cognition.

Finally, we argue that the degree of abstractness of a concept is determined by the variability of its referents, which probably correlates with the aforementioned distance. As an example, consider the visual domain; the lowest variability occurs for physical objects as a whole (i.e. the concept refers to the whole object, not its parts or properties). The most concrete concepts only have one reference (e.g. the moon). Then, there exists the basic level of categorization that provides a vast number of referents with relatively low variability (e.g. a dog). Next, going toward a superordinate level, the variability starts to increase (e.g. a mammal). The same applies to object parts, for instance, body parts that apply to morphologically distinct organisms (e.g. the head). This line of thinking is similar to Pulvermüller (2018) who also points to a higher variability of referents for abstract concepts (such as beauty) compared to concrete concepts (eye). He also attributes a crucial role of words (i.e. the linguistic system) in establishing the abstract concepts (by binding operation), which applies, in our view also to the superordinate level of categorization of concrete objects. The variability of referents of abstract concepts, typically providing relevant situational contexts, is in general very high, and most probably even within this domain one could demarcate a certain hierarchy.

The view on graded abstractness has emerged in other recent works, drawing on evidence from psychology, neuroscience and computational linguistics. One empirical study revealed systematic quantitative differences between concrete and abstract words, represented in latent semantic space (Troche, Crutch & Reilly, 2014), obtained from subjective assessments. The authors investigated word meaning as originally distributed in multidimensional space using hierarchical cluster analysis. Participants rated 400 English nouns across cognitive dimensions (e.g., polarity, ease of teaching, emotional valence) and these vectors were then projected onto three latent factors, corresponding roughly to perceptual salience, affective association, and magnitude. The visualisation of words in this latent space showed that abstract and concrete words overlapped in their topography but also differentiated themselves in semantic space. In addition, the method allowed the authors to represent the degree of concreteness of each word (on Likert scale). This obviated the need for an artificial dichotomy by treating all psycholinguistic variables as continuous.

Hill, Reichart and Korhonen (2014) also point to a gradual contrast in patterns of organisation along a continuum from concrete to abstract concepts. The terms association and similarity refer to the ways the concept pairs (onion, knife) and (onion, carrot) are related: Onion is said to be (semantically) similar to carrot and associated with (but not similar to) knife. Repeated analyses using existing word corpora and human judgments have shown the following (see Hill et al. and references therein): (1) abstract concepts are mainly organised according to association, whereas concrete concepts are organised according to (semantic) similarity (2) abstract words have more, but weaker, associations with other words, (3) abstract words have more symmetric associations than concrete words. This analysis supports a gradual contrast in patterns of organisation along the continuum.

Chatterjee (2010) takes a neuroscientific approach to graded abstractness. He refers to cases of patients with motor deficits whose conceptual understanding remained intact, suggesting a disembodied level of understanding. In our view, this is reminiscent of the distinction between deep, sensorimotor understanding and shallow, conceptual understanding, as articulated by Rizzolatti and Sinigaglia (2010) in the context of the mirror neuron theory. Even in the case of healthy individuals, such a distinction appears reasonable, when considering motor actions being in one’s own motor repertoire (e.g. nail hammering), compared to those beyond it (e.g. eagle flying). Drawing on his own work on spatial cognition and language, Chatterjee (2010) also proposes three functional-anatomic axes in the brain that could be related to graded abstractness. These are lateral axis that could reveal laterality differences in the processing of sensory and motor attributes as they relate to concepts, ventral–dorsal axis that appears to correlate with a shift from rich conceptual information to more schematic relationships, and centripetal axis that goes from sensory and motor cortices to perisylvian language cortices.

Neuropsychological literature has converged to three hypotheses how concrete and abstract words differ quantitatively, as reviewed by Hoffman (2016): (1) They differ in their representational substrates, with concrete words depending particularly on sensory experiences and abstract words on linguistic, emotional, and magnitude-based information; (2) Abstract words place greater demands on executive regulation processes because they have variable meanings that change with context; (3) The relationships between concrete words are governed primarily by conceptual similarity, while those of abstract words depend more on associations.

A global picture of knowledge organisation in the brain has been proposed by Taylor et al. (2015) who performed a large-scale meta-analysis of fMRI data. The hypothesis was tested, using formal methods based on a new cortical graph metrics (network depth) that regions deeper in the brain (i.e. remote from the sensory inputs) represent more abstract functions. Data-driven analyses defined a hierarchically ordered connectome, revealing a related continuum of cognitive functions. The data were collected from participants performing cognitive tasks, such as sorting words or phrases according to the estimated degree of abstractness. Taylor et al. conclude that progressive functional abstraction over the network depth may be a fundamental feature of the human brain.

Analogical approaches in computational linguistics take advantage of huge linguistic corpora. The fact that covariation in the world is reflected in the structure of language (Glenberg & Mehta, 2009) has been repeatedly confirmed in comparing semantic similarities between words with human judgements. We can also assume that semantically similar words tend to have similar degrees of abstractness. Hence, in the linguistic representational space there could exist a nonlinear concrete–abstract dimension (or dimensions) along which the words could be organised. Alternatively, rather that going deep into semantic space via word embeddings (which is used in methods such as Latent Semantic Analysis, Global Vectors, Word2vec and Bidirectional Encoder Representations from Transformers, or BERT), one could remain on the surface level (see the arguments in Louwerse, 2011) and look at word covariation when searching for word abstractness. The abstractness of a word could be estimated from characteristics of words associated with it (by co-occurrence), where we could expect that word abstractness would be correlated with a higher percentage of abstract words associated with it.

Huge linguistic corpora provide rich extractable information, but despite the wide use of distributional methods in the natural language processing field, there remains the problem of symbol grounding (Harnad, 1990). By focusing merely on the form one cannot in principle retrieve meaning, without additional reference to the world, not even for BERT models (Devlin et al., 2019) whose performance is otherwise truly impressive (Bender & Koller, 2020). Similarly, although distributional models display a number of interesting properties (Boleda, 2020), their limitation as an account of cognition seems to be their accuracy. For instance, an extensive study (Binder et al., 2016) questions the ability of corpus-based word vectors (LSA) to accurately reflect the similarities between concepts, when they were compared to brain-based 65-dimensional vectors of attributes (comprising sensory, motor, spatial, temporal, affective, social, and cognitive experiences). Using both methods, the word vectors were constructed for 434 nouns, 62 verbs, and 39 adjectives, by averaging the responses of 1743 participants, and their study revealed better within- versus between-category separation than representations derived from distributional (LSA-based) text analysis.

The above references provide rich evidence that abstractness has its correlates, be it psychological features (measured by subjective assessments of various words), neural features (based on brain imaging studies), or linguistic features (revealed by corpus analyses).^4^ In each case, the relation between these referents and one (or both) of the two pathways of grounding discussed herein.

## 6. Conclusion

In this paper we argued that, in principle, all cognition is grounded in experience via two different pathways: primarily directly through the body (which includes sensorimotor interactions with the environment, interoception, and emotions), and, as a secondary pathway, indirectly by means of communication (linguistic and social processes). Departing from a dichotomous view, we suggest abstractness can be seen as a graded phenomenon, so that abstractness–concreteness is reconceptualised as a continuum that can be related to various psychological, neural and linguistic features. As a novelty, we illustrated the grounding of abstract concepts by three case studies covering the spectrum of abstractness levels (body-specificity, abstract verbs, maths). We also reviewed neurodevelopmental insights that support differences between these two pathways of grounding. In this way, we extend an earlier review by Borghi et al. (2017) that had begun to summarise the crystallisation of the continuum view on abstractness. Crucial open questions we have raised include a more detailed neuroscientific account of how abstract conceptual knowledge depends on embodiment (direct grounding) and the sensorimotor brain systems. At the same time, we have also challenged opponents of an embodied view to offer alternative explanations to the results of the case studies and related literature we reported here. One strength of our view in this respect is that it integrates different perspectives on language as one type of grounding that is itself grounded and embodied. Major challenges are also faced by the computational approaches to abstraction, where the examples so far have been limited to toy cases with small vocabularies (Cangelosi & Stramandinoli, 2018). One promising approach is offered by large-scale distributional methods in natural language processing, which remain an exception in their field and will probably significantly contribute to acquisition of abstract concepts in computer models using the second pathway, enriching the primary sources of concrete and abstract knowledge resulting from interactions with the world.

## Footnotes

Dove’s dis-embodied view on language differs from traditional disembodied view (e.g. Mahon and Caramazza, 2008) which does not rely on sensorimotor simulations in linguistic processing of concepts, but rather postulates disembodied processes using amodal symbols.

The conceptual metaphor theory is not universally accepted. For instance, McGlone (2011) argues that it is ambiguous, not parsimonious, it suffers from “homunculus problem” and employs circular reasoning. Mahon and Caramazza (2008) present more general criticism, defending the symbolic nature of abstract knowledge.

As a limitation, it should be acknowledged, direct mapping applies to (some) concepts related to motor actions, so it does not provide an overarching principle, especially when it comes to abstract concepts.

Interestingly, graded abstractness has also been recently identified as one of these four dimensions that define the notion of another high-level concept: consciousness (Jonkisz, Wierzchoń, & Binder, 2017).
